# Clinical Lecture on Anæmia and Bloodletting

**Published:** 1862-04

**Authors:** Thomas K. Chambers

**Affiliations:** Lecturer on Systematic and Clinical Medicine


### SELECTED.

### CLINICAL LECTURE ON ANÆMIA AND BLOOD-LETTING.

*Delivered at St. Mary's Hospital, Nov. 14th and 28th, 1861.*

By THOMAS K. CHAMBERS,
*Lecturer on Systematic and Clinical Medicine.*

Gentlemen: You will all remember the corpse like pallor, made more conspicuous by red hair, of a girl, admitted this day fortnight into Victoria Ward. She smiled courteously, but was quite unable to rise from her bed. Her history is as follows:—

Margaret C., now aged 20, seems to have had very good health in general, as is shown by her remembering that she had such an unimportant ailment as a pain in the right side when she was a school girl of seven years old. She seems to have been carefully brought up by a step-father in a higher class of life; but three years ago she lost him, and had to go into service as a housemaid. For this work she was hardly strong enough, and, perhaps, too tenderly educated, and after eighteen months' trial she gave it up, and was apprenticed to a Berlin-wool shop. In this place her mental superiority was apparently recognized, for she quickly became forewoman, with three girls under her, in a shop at Maidstone. She felt this responsibility a good deal, and also thought the closeness of the shop did not suit her, though it did not seem to make others ill. However, she retained a high, bright color in her face, for which she seems to have been somewhat admired, till nine months ago, when she began to lose it, and in a few weeks became as waxlike in hue as she is now. At first her appetite was large, and she always seemed in want of food; but after three months it failed, then ceased entirely, and she took a disgust to food. She had a good deal of pain in the epigastrium and to the left side of it, and also palpitations and pain of the heart. Three months ago she spat up some blood, and had a little cough, which frightened her sadly Three times during the nine months she has had attacks of low spirits, with crying, but does not appear at all hysterical now. The catamenia always were quite regular and sufficient till the commencement of the anæmia nine months ago, when they began to get scantier and scantier, and at last ceased entirely. The urine is pale and watery, the stools scanty and steadily rare; but there is no sudden gush of bulky stools, diarrhœa alternating with constipation, or other indications of accumulation of feces in the intestines.

She expands her chest perfectly, and there are no abnormalities to give rise to a a suspicion of pulmonary tubercle, at all events in such a quantity as to cause anæmia. There was a soft and systolic murmur in the heart when she was agitated at first admission; but it went away after she had rested in bed five days.

First, now, for the name by which I have already designated this patient's disease. Anæmia, or "bloodlessness," means in scientific language a deficiency of the red disks in the blood. The word has been objected to because it has been supposed to imply etymologically that there is a deficiency in the actual quantity of circulating fluid, of which, indeed, there is no proof. And "Spanæmia," or "thinness of blood," has been proposed in its stead. Such accuracy would be highly commendable, if it were only accurate; but in truth the fact of *thinness* does not describe the essential nature of the disease; for the specific gravity of the blood might be raised as high as you like, but if you did not restore red blood-disks, nothing would be gained; the morbid state would still exist. But, in reality, there is no occasion for fault-finding. Anæmia, by the analogy of Greek etymology, does not mean deficient quantity of blood, but deficient quality, just as in Aristophanes *aprosopos* does not mean a man "without a face," but "with an ugly face," *anarithmos* means "difficult to count," and so on. I shall theerefore contentedly use the term to include all cases in which the blood-disks are beneath the normal proportion.

Anæmia is found during life in a great number of the organic changes of tissues which you see in museums and lectures on morbid anatomy, and may discover by diagnosis. In other cases of equal importance and prominence it is absent. Very frequently, too, you find it in an extremely high degree in cases where you can discover no organic change in the solids at all, and where, from the transitory nature of the bloodlessness, there is reason to conclude that such organic change really does not exist. Under this last category comes the patient who is the occasion of my present Lecture.

To understand how it is that so many causes are followed by the same effect, and by an effect by no means proportioned to the general importance or want of importance of the cause, you must reflect upon the true relation which the blood bears to the rest of the organism. It is in the same position as a great thoroughfare in an important town. Very little trade, and still less manufacture, is carried on in the street itself, yet from the nature, the number, the pace, and other characteristics of the vehicles and people which pass, you may form a pretty shrewd notion of the commercial prosperity of the population. A foreigner standing in Cornhill and viewing the steady quick pace, and active, careful, yet healthy faces of the many-classed by-passers, the well-packed loads of the vehicles, and their varied yet subdivided contents, cannot fail to see that he is an industrious well-to-do nation. But it is not the mere fact of the crowding that makes him say so, for last Saturday he would have seen a greater crush at the same place though that was only in consequence of all trade being suspended for Lord Mayor's-day. And at Naples, the lazaroni and pickpockets who block up the pavements are evidences that trade is not only suspended, but prevented, by a dangerous horde of villains. So, in the blood, the physician traces proof how constructive metamorphosis (the city's manufacturing industry), destructive metamorphosis (its consumption), and effective life (its social happiness) are carried on.

The traveler must not be deceived by an idle multitude in one spot, in estimating the strength of the population, nor must we set down local congestion as proof of excess of blood. In both cases, experience shows we have strong presumptive evidence of a deficiency.

Neither must a mere bustling throng be reckoned as industrious citizens. There are cases where a large amount of solid matter, even where a large amount of red disks, adds no more to the usefulness of the circulating fluid than the lazaroni to Naples, and which are, therefore, as far as treatment is concerned, really in a condition of anæmia. Of these cases I will speak at a future opportunity.

But though crowds are no evidence of sound political health, yet it is certain that deserted streets prove the contrary. So anæmia, or deficient redness in the blood shows a deficiency of life in the ministers to that redness; either the supply of food is too small, or its assimilation is defective, in both cases either absolutely or relatively, to the existing demand.

In many instances it is easy enough to lay the finger upon the instrument of life which is to blame. We can detect without difficulty the causes at work—starvation, which anybody can understand, leads to an absence of the organic matters made out of food; disease of stomach, in which the aliments are not prepared for assimilation; disease of liver and duodenum, producing the same result; disease of intestines, or their glands taking up no adipose matter especially, and so preventing cell growth; disease of the spleen or lungs, which physiological experiments, independent even of our observations of morbid phenomena, show to be answerable for the formation of new blood disks in a way yet unknown; mental derangement, care, disappointment, which so readily arrest the activity of the assimilating viscera; these agencies, and many more, are readily comprehended as causes of anæmia. But there are a considerable number of cases where nothing tangible of this sort is to be made out, yet where the paleness of the blood seen in the face, lips, tongue, or in a drop taken from a pricked finger, and evidenced by the faintness, weakness, palpitation, anasarca, amenorrhœa, etc., are even more marked than where demonstrable lesion is to be found. So it is in the present instance. The young woman's history gives no reason to suspect any organic disease of the lungs or other organs, and the functions of life were fairly performed till she began to get pale and languid nine months ago. The want of red blood, which we look upon as the important feature in her case, attracted her attention also particularly, as she had previously had a fresh, high color. Then, after an interval amply sufficient to enable us to separate cause and effect, come the symptoms which I wish to notice as the consequences of anæmia. Causes, no doubt, they are in some instances, but here consequences. I mean the loss of appetite, impeded circulation, cessation of menses, hemorrhage from respiratory mucous membrane, and hysteria in a person unaccustomed to it.

The only explanation she can give of her loss of health is her having been employed in a shop less ventilated than she had been accustomed to, and having the responsibility of the concern thrown upon her. Alone neither would have been sufficient, as the shopwomen under her do not appear to have suffered from the air; while, on the other hand, women in retail business are not as a rule anæmic. But still I think that both together may perhaps be fairly saddled with the blame, for whilst the increased mental labor was increasing metamorphosis, the greater demand was not responded to by greater supply, but, on the contrary, assimilation was checked by the even moderate unwholesomeness of the respired air.

Of course, the not being able to trace deeper, the anatomical cause arises from the imperfection of our knowledge, but it does not arise from neglecting to apply such knowledge as we possess to practical medicine. If we were to make an autopsy of this patient instead of curing her, we should in all probability find no more lesions in any of the tissues capable of accounting for the disease exhibited in the blood than we have already found. A fortnight ago Dr. Gull, Mr. Malton, and I examined the body of a gentleman who had died at forty-six of anæmia, and made separately microscopical investigations of portions of the several viscera. Nothing abnormal could we find in any part. The typical healthiness of all tissues was very remarkable in a man of that age. There was not even a single adhesion of the pleura. I mention this in order that you may not lament the opacity of your patient's bodies, or suppose yourselves likely to learn how to treat them better if you could see their insides.

Anæmia, without obvious organic lesion, when properly treated, is a very curable condition, and this should still further reassure you, that you miss nothing by not being able to study its post-mortem pathology. For transitory and curable states leave but little foot-prints behind them for morbid anatomists. In a great majority of cases they depend upon the mucous membrane, of all the tissues in the body the one most affected by mortuary changes.

To the mucous membrane I am disposed to attribute the condition in which we find our present patient. The two circumstances to which I have traced the illness both act directly or indirectly on the tissue. The mental exertion involved in an unusual responsibility thrown on a conscientious person would arrest the action of the involuntary muscles which carry along the mass of food through the alimentary canal. You know well the time your food is in leaving the stomach if you are called to an important midwifery case just after a hearty meal; and several commercial and literary men have complained to me of attacks of vomiting (that is, temporary paralysis of the stomach), when they took dinner alone, and so were apt to let the mind dwell deeply on some interesting subject; and they have told me in wonder that they could dine out and eat and drink all sorts of rich things with impunity. They did not seem aware of the physiological value of frivolous conversation. At the same time that the moral causes thus impeded digestion, the unwholesomeness of the air in the close shop poisoned the mucous membranes, diminishing their vitality and causing them to be abnormally covered with a thick layer of mucus. Remember that, in spite of their name, it is not the business of mucous membranes tosecrete mucus; the more perfect is their condition, the more favorable are the surrounding circumstances, the less they do so. From many persons' lungs not a drachm of expectoration is thrown up in a month, and the vast surfaces of the intestines and bladder are equally innocent of even microscopic traces of mucus in the typical health we desire to experience. It is only when the presence of some material agent diminishes their vitality that the mucous membranes exhibit on their surfaces that peculiar substance whence they take their appellation. And the greater the diminution of life, the greater the secretion; a slight cold in the head will be accompanied by slight catarrh, a severe one by excessive catarrh; and the nearer the approach to death, the nearer it is, so that the death rattle, or overpowering collection of mucus in the bronchi, is a popular warning that all is over. Be careful not to look upon mucous secretion as augmented life; it is in fact a partial death.

Well, the poisoning air having covered these slowly moving mucous membranes with a thick tenacious coat, the entrance of alimentary substances into the veins and absorbents was impeded, and our patient starved in the midst of plenty. So all the usual signs of starvation followed. First, hunger—by no means a constant accompaniment of chronic deprivation of food, yet sometimes present as here; then anorexia, a much more frequent phenomenon; then paleness, languor, weariness, and pain in the stomach; then anasarca, and, in short, the other more marked symptoms of anæmia.

You may observe that the loss in those constituents of the body, which are of a nitrogenous chemical composition, is more marked than that in the hydrocarbonaceous fat. The reason is, partly, that the destruction of adipose vesicles is somewhat concealed by the saturation of the tissue with serum, which gives it a false plumpness—partly, that fat, being absorbable without much, if any, alteration, is easier taken up than fibrin or albumen which require a chemical solution before they can be absorbed. So that though starved, our patient looks but little emaciated.

All that I have said before, of course has for its end the treatment. My aim in anæmia is to introduce as quickly as I can the largest possible amount of: 1, nitrogenous food; 2, iron; 3, chlorine. When I say "introduce" I do not mean "throw in," or get swallowed, but assimilated in the system.

As regards the first, it is obvious that if I had written down ever so many "ordinary diets," a patient to whom the very sight of food was an abomination, would have gained nothing by it; she would simply have gone without. I directed, therefore, no meals at all, and no solid food, but a cup of milk with some lime-water in it, to be given as medicine every two hours, and a pint of beef-tea in small, divided doses during the day. After two days she managed an egg also daily, and after twelve days of gradual additions of this sort, you will find her on full allowance of mutton chop, porter, beef-tea and milk.

Iron is required to supply the new growth of red disks which we hope for, with their metallic constituent. You cannot get it into the system in any way so quickly as the mistura ferri composita of the London Pharmacopœia. Large doses of the more soluble salts have an action on the mucous membranes which not only prevents them being taken up, but also arrests the digestion of food. Evidence of the latter is found in loss of appetite and feverishness, and of their own rejection in the blackening of the stools much sooner than by the form I have approved of. So in spite of the elegant preparations which are constantly put before us, as recommended by their solubility, such as the chloride, acetate, citrate, phosphate and other salts of iron, I prefer the unchemical mixture. It seems as if the carbonate which is preserved from decomposition by the sugar, and the finely-divided oxides diffused through the thick liquid were peculiarly easy of solution in the water saturated with salts and carbonic acid, which (and not pure water) we must remember is the solvent to be considered.

I have found that some cases which did not improve so quickly as I could wish under the above treatment, made a sudden start of improvement when to it was added the administration of chlorine in the form of warm hydrochloric acid baths. More iron is taken up—the blackening of the feces ceases, and therefore perhaps it may be that the presence of more acid in the system attracts more of the metal. But in a few cases I tried for experiment the hydrochloric acid baths alone, and even then it was beneficial, seeming to confer muscular strength like what are commonly called tonic drugs. I cannot but think, therefore, that it supplies a distinct want in the system, that it is a directly restorative medicine in anæmia.

Nor is it difficult to make this empirical observation accord with rational pathology. In anæmia the blood is more watery than natural; the proportion is deficient, not only of organic matters, but of salts. Chloride of sodium is the most important of these, and the supply of one of the constituents of this material we may personally imagin is an aid to the renewal of life, which is the end of all medication.

Besides the above named medicines, you will see, I have ordered Pil. aloes cum myrrha, gr. iv, omni nocte sumenda. Now, do not suppose that this is ordered merely as a purgative, and that any other purgative would do as well. On the contrary, most purgatives do harm in anæmia. Gamboge, castor-oil, sulphate of magnesia, colocynth, mercury and several others, which produce serous elimination and augment secretion generally, would do harm just in proportion to their activity. It seems established by the experiment of making them as purgatives when injected into the circulation, that their soluble principles have a destructive agency over the blood; whereas the soluble alkaloid in aloes (aloine) is, in fact, a bitter tonic, and the purgative power of the drug resides in its insoluble resin.* Its action is very slightly eliminative—in moderate doses it only slightly augments the solid brown excreta of the colonic glands, and produces feces feculent in smell and of consistent form; whilst at the same time it restrains, by its bracing bitter, the formation of mucus, as you may clearly see by its action on moist piles, how it dries them up and make them smart. And by the more vigorous peristaltic action and by the solid mass passed along the gut, the already existing mucus is cleared away. Aloes, therefore, is employed strictly as a clearer of the intestinal, especially of the colonic, membrane. It is joined with myrrh, partly to divide it minutely, and make a small dose go further; and partly to get the advantage of the extra resin.

November 28. A fortnight ago I lectured about an anæmic patient. She was then showing a tendency to lose her title to the name, and now she certainly cannot claim it, and has earned our confidence in the statement that her natural hue is rosy.

* "Headland on the Action of Medicines," p. 331; and Robiquet in *Journal de Pharmacie*, April, 1856.

She leaves the Hospital to-day, having manufactured enough red disks to colour her blood throughout very sufficiently.

What amount of manufacturing industry does this show? Let us reckon. She weighs 8 stone, or 1792 ounces; of this 2-7ths, or 512 ounces is blood; and of this blood 133-1000, that is to say, 60 ounces should be red globules. Now the analyses of MM. Andral and Gavarret show that in cases of anæmia of at all a marked character (as this was), we may expect, at least, three-quarters of the red disks to disappear, so that when she came into the Hospital it may be fairly assumed that she did not possess above 15 ounces; and now I think with equal fairness she may be assumed to have got up to 45, which is conceding that she still wants a quarter of perfect health. By this reckoning she must have made 20 ounces of red blood disks; that is, the most important organic constituent of upwards of 150 ounces of blood, in a month!

Mark the power of renewal which the human body has under favorable circumstances, and learn from this not only the curability of anæmia when it is a disease, but also the facility of repairing artificial loss of blood when it is employed as a remedy. It has been the fashion lately among certain medical declaimers to paint the Physician who draws ten or twelve ounces of blood from the arm as a deadly villain, who necessarily *ex vi termini* takes away "the life," or that which cannot be replaced. Not only pill-dealers and quacks have raised this outcry, but it has been joined in by some whose knowledge of physiology ought to have taught them better. It ought to have taught them the fallacy of the popular notion and the scientific argument by which to refute it. You will clearly perceive from the calculations through which I have taken you, that by proper management no loss is so easily repaired, and that if he saves his patient two nights' sleeplessness or pain, the price of a venesection is well spent.

Only note this, that *if the loss is to be repaired, the means of repair must be given.* When I bleed, you will observe that I take down the diet card and accommodate it to the circumstances, being very careful that the patient has the wherewithal to replace the globules I am detracting. I supply with one hand what I am taking away with the other. I begin to cure the artificial anæmia, which I feel myself called upon to produce, at the same time that I am producing it. "Blowing hot and cold," you will say. Precisely so—that is what I intend. I blow cold with my bleeding, not for the sake of blowing cold, but because it is the inevitable accompaniment of the remedy.

I employ the remedy not to produce anæmia, but for other quite different purposes which I think are worth the cost. And I blow hot to compensate as well as I can for the evil I think it desirable to do, on the principle

"Necesse est facere sumptum, qui quærit lucrum."

I do believe the sad effects of the excessive venesection of our fathers, which, with justice, have been thrown in the teeth of the Medical Profession, was due quite as much to starvation as to the bleeding. I have a most vivid and painful recollection of seeing, when I was a student in Paris, M. Chomel and others treating pneumonia. I could not at first understand why in France so much more marked, and, in my opinion, so much more deleterious, effects were produced by the venesection than in England. At that period we had at home ample opportunities of seeing blood-letting practised; but I never saw such prostration produced by it at St. George's as I did at the Hotel Dien. Then I noticed that the order for "*Saignee*" was accompanied by "*Diete absolue.*" I almost doubted my knowledge of French, and was obliged to ask several of the bystanders before I could believe that this meant an *utter deprivation of all food!* There was an instantaneous explanation of the comparative toughness of my countrymen; for never in our worst days did we carry the Sangrado practice so far as that. We did not give food enough, perhaps, but we never commanded that it should be intentionally kept out of our patient's way.

The bad practice of starving and bleeding at the same time, took its rise from the errors of Allopathy. In this system a disease is an enemy to be overcome—a something *to be combated by* an agent which is as opposite to it as possible. Bleeding was found by experience to be useful in certain morbid states; therefore it was useful in virtue of its opposite effects. Anæmia and depression of life, are the most constant effects of bleeding; therefore anæmia and depression are the benefactors to be sought for, and whatever aids bloodletting in producing anæmia and depression, is a good companion to it. It is unnecessary to say, that of course starvation was the first agent thought of, adopted *à l'outrance* by the logical French, and with more hesitation by our fortunately illogical countrymen. The abuse has brought about a reaction; and that treatment which was considered at one time so specific that its gravest faults were viewed as virtues, now runs a risk of being denied *all* virtue because of its avoidable faults.—*Med. Times and Gazette, Jan.* 11, 1862.

